# Evidence for ~12-h ultradian gene programs in humans

**DOI:** 10.1038/s44323-024-00005-1

**Published:** 2024-08-09

**Authors:** Bokai Zhu, Silvia Liu, Natalie L. David, William Dion, Nandini K. Doshi, Lauren B. Siegel, Tânia Amorim, Rosemary E. Andrews, G. V. Naveen Kumar, Hanwen Li, Saad Irfan, Tristan Pesaresi, Ankit X. Sharma, Michelle Sun, Pouneh K. Fazeli, Matthew L. Steinhauser

**Affiliations:** 1 Aging Institute of UPMC, University of Pittsburgh School of Medicine, Pittsburgh, PA USA; 2 Pittsburgh Liver Research Center, University of Pittsburgh, Pittsburgh, PA USA; 3 Division of Endocrinology and Metabolism, Department of Medicine, University of Pittsburgh School of Medicine, Pittsburgh, PA USA; 4 Department of Pathology, University of Pittsburgh School of Medicine, Pittsburgh, PA USA; 5 Neuroendocrinology Unit, Division of Endocrinology and Metabolism, Department of Medicine, University of Pittsburgh School of Medicine, Pittsburgh, PA USA; 6 Center for Human Integrative Physiology, University of Pittsburgh School of Medicine, Pittsburgh, PA USA; 7 Department of Statistics, Kenneth P. Dietrich School of Arts and Sciences, University of Pittsburgh, Pittsburgh, PA USA; 8 Division of Cardiology, Department of Medicine, University of Pittsburgh School of Medicine, Pittsburgh, PA USA

**Keywords:** Cell biology, Diseases

## Abstract

Mice and many marine organisms exhibit ~12-h ultradian rhythms, however, direct evidence of ~12-h ultradian rhythms in humans is lacking. Here, we performed prospective, temporal transcriptome profiling of peripheral white blood cells from three healthy humans. All three participants independently exhibited robust ~12-h transcriptional rhythms in molecular programs involved in RNA and protein metabolism, with strong homology to circatidal gene programs previously identified in Cnidarian marine species.

Our understanding of biological rhythms is expanded in part by alternative, non-circadian rhythms discovered in lower organisms. Coastal marine organisms, such as the sea anemone *A. diaphana*, exhibit ~12-h ultradian rhythms, entrained by the twice daily movements of the tides^1^. In mice, ~12-h rhythms of gene expression involved in mRNA, protein and lipid metabolism are established by an XBP1-dependent oscillator independent of the circadian clock or cell cycle^2,3^. Oscillation of some physiological metrics at an ~12-h interval in humans suggests the possible existence of 12-h rhythms in humans^4–9^. However, evidence for ~12-h rhythms at the molecular level in humans is lacking.

We studied three healthy males (Table 1) who self-reported a regular nighttime sleep schedule and did not engage in nighttime shift work or other sleep-disrupting activities and who had a body mass index between 23.3 and 24.4 kg/m^2^. Participants were admitted for a 1-day acclimatization period prior to 48 h of blood sampling at a 2-h sampling interval (Fig. 1A), a frequency that allowed detection of oscillations with periods of 6 h or longer with high confidence^10,11^. The protocol reflected our aim to sample with sufficient frequency and duration to detect ~12-h rhythms within individuals. We also sought to mimic free-living conditions by providing a diet similar in caloric content to their typical intake and encouraging maintenance of their routine sleep/wake cycles. Overnight, blood was collected via a long intravenous line from outside the room to avoid exposure to light or sleep disruption.

**Table 1:** Participant characteristics.

	Participant 1	Participant 2	Participant 3
Age (years)	20	22	28
Body mass index (kg/m^2^)	23.3	23.7	24.4
Vitamin D (ng/mL)	85	26	22
Glucose (mg/dL)	73	84	74
BUN (mg/dL)	10	18	14
Creatinine (mg/dL)	0.9	1.0	0.9
AST (U/L)	22	41	22
ALT (U/L)	10	28	22
TSH (mIU/L)	1.306	1.48	0.803

**Fig. 1:**
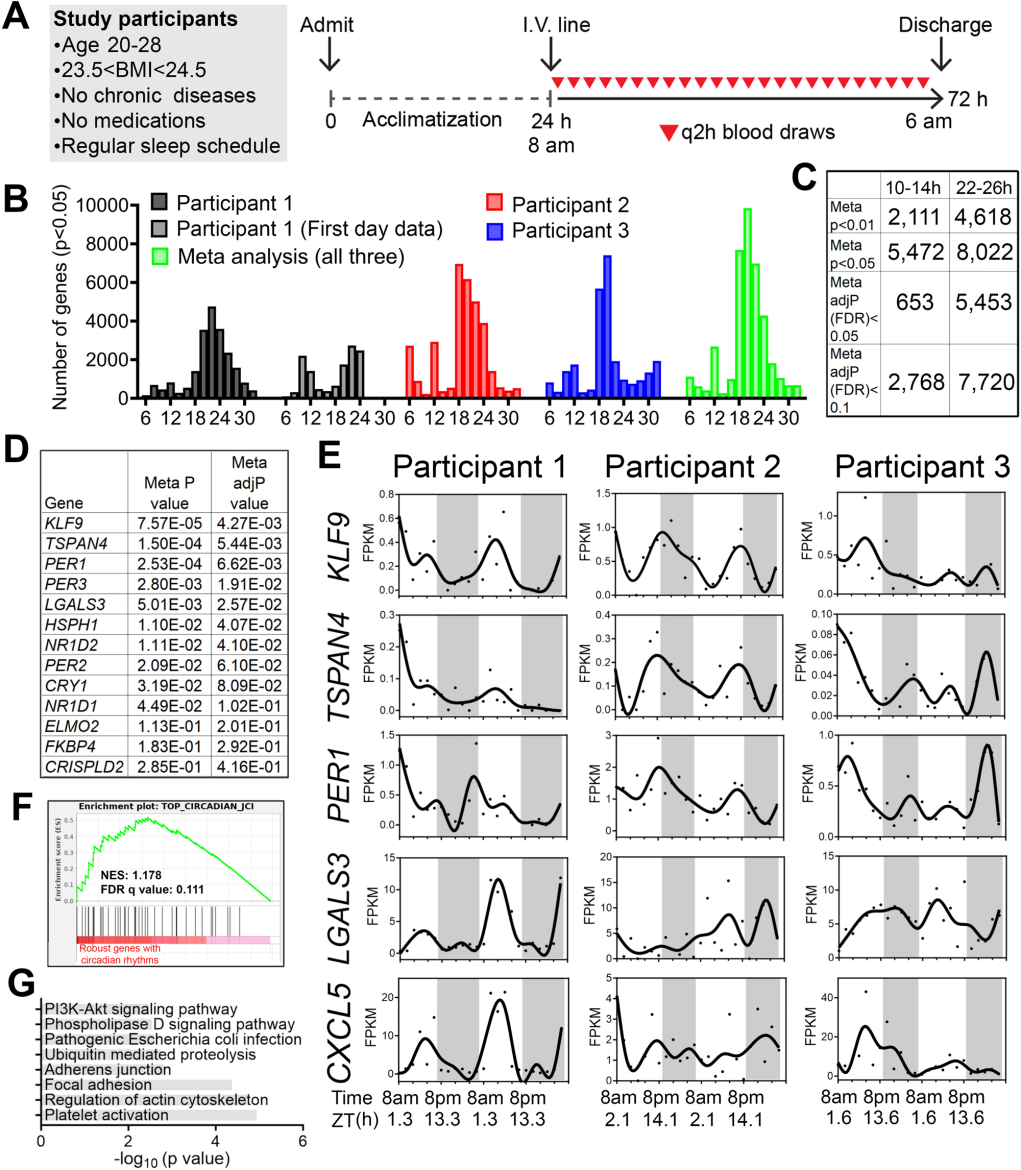
Identification of inter-individual variability of ~24-h and ultradian rhythms of gene expression in peripheral white blood cells of humans. **A** Human protocol and study schematic. **B**–**D** Histograms showing the period distributions of all rhythmic genes uncovered from the three participants (with *p* value < 0.05) as well as the period distribution of rhythmic genes calculated by the meta *p* values (meta *p* < 0.05). For the first individual, periods calculated from all 48 h of data, or the first 24 h of the dataset were both shown. **C** Table summarizing the number of ~24-h and ~12-h genes with different statistical cut-offs. **D** Table listing the meta P and meta adj-P (FDR) values of thirteen ~24-h genes previously used as training datasets for predicting ~24-h phase from human blood samples^15^. **E** Raw temporal expression profile (dot) and spline fit (solid line) of five of the thirteen ~24-h genes. **F** Gene set enrichment analysis (GSEA) showing enrichment of a previously identified set of top forty robustly-cycling ~24-h genes in human white blood cells^15^ with robust ~24-h rhythmicity in our study. **G** GO analyses of top KEGG pathways for ~24-h genes with meta adj-P (FDR) less than 0.01.

We performed bulk mRNA-Seq in buffy coat fractions prospectively collected at 2-h intervals for 48 h (24 samples/participant) (Fig. 1A) (Table S1). To identify genes cycling with a period ranging from 6 to 32 h, we applied the RAIN algorithm to each participant’s temporal transcriptome^12^. Compared to alternative methods like JTK_CYCLE, RAIN detects rhythms with arbitrary waveforms and therefore more robustly uncovers ultradian rhythms^11,13,14^. As shown in Fig. 1B, we observed inter-individual variability in the number of genes cycling at different periods. Specifically, the first individual exhibited dominant oscillations cycling at the period between 20 and 24-h, with a secondary population detected at the ultradian periods of 10–12 h. These 10–12-h oscillations were more evident in the first 24 h, with dampening of ultradian rhythms on the second day, despite there being no obvious physiological perturbation or difference between the first and second day of sampling (Fig. 1B). Aside from oscillations between 18 and 24 h, the second participant exhibited ultradian rhythms cycling at periods of 6 and 12-h (Fig. 1B). The third individual also exhibited oscillations cycling at 6-h, 12-h, and at ~20- h with fewer genes observed cycling at 24-h (Fig. 1B). The observed inter-individual variability of transcriptomes cycling at ~24-h is not surprising, as inter-individual variations in the number, period, phase and amplitude of circadian genes were also observed in a prior study where participants were maintained in a semi-recumbent position under dim light and constant temperature and humidity during 40 h of sleep deprivation (Fig. S1A, B)^15^.

To increase statistical power to detect oscillations common to all three individuals, we performed a meta-analysis and generated a combined *p*-value for each gene at periods from 6–32-h using Fisher’s method. This method has been extensively used in medical and genetic research to merge results from independent tests, each with the same null hypothesis^16^. In our study, each participant was distinct and studied at a different time, however the null hypothesis was the same: the absence of rhythms. Using combined p-values (herein referred to as meta *p* values) with an alpha of 0.05, we observed two major populations of oscillations cycling at the periods of 12 and 18–26-h, with the largest number of genes observed cycling at the period of 20-h (Fig. 1B). Using the Benjamini-Hochberg procedure to further adjust for the false discovery rate on meta *p* values (meta adj-*P* value or FDR), we uncovered 653 genes with periods between 10–14-h and 5453 genes with periods between 22–26-h genes with an FDR less than 0.05 (Fig. 1C) (Table S2).

To determine the soundness of our RNA-seq dataset, we compared 22–26-h cycling genes identified in our study with those uncovered by Wittenbrink and colleagues^15^. In that study, thirteen genes exhibiting robust circadian expression in peripheral white blood cells were utilized as a training gene set to predict the circadian phases from single time point blood samples^15^. In our study, seven and nine (of the core set of 13 genes) exhibited meta adj-*P* values less than 0.05 and 0.1, respectively, including canonical circadian clock genes *PER1*, *PER2*, *PER3*, *NR1D1*, *NR1D2* and *CRY1* (Fig. 1D, E). Gene set enrichment analysis (GSEA) further indicated that the forty circadian genes that cycled in at least 50% of the human participants in the study by Wittenbrink et al.^15^, also exhibited oscillations cycling between 22–26 h with low meta *p* values in our study (Fig. 1F). These genes were linked to pathways of platelet activation, regulation of actin cytoskeleton and adhesions (Fig. 1G). Collectively, these results demonstrate the possibility of detecting biologically relevant rhythms in human gene expression under conditions designed to mimic free-living.

Oscillations cycling close to a 12-h period were the second most abundant in all three individuals after the 22–26-h population (Fig. 1B). Using meta adj-*P* values (FDR) as cut-off, we identified 653 and 2768 ~ 12-h genes (with period between 10–14-h) with meta adj-P (FDR) less than 0.05 and 0.1, respectively (Figs. 1C, 2A). As observed with 22–26-h genes, inter-individual variability was also observed for ~12-h ultradian rhythms. In the first and second participants, the acrophases (time of gene expression peak) were at 8 am and 8 pm, while ~12-h rhythms in the third individual peaked around 4 am and 4 pm (Fig. 2A). Examples of genes that robustly cycled at an ~12-h period in all three individuals include *MYDGF*, *ICMT*, *RNF7*, *TCEB1*, *MFF,* and *OXA1L* (Fig. 2B). *MYDGF* encodes an endoplasmic reticulum (ER)-localized protein with secreted forms from monocytes/macrophages that promote tissue repair in a murine model of myocardial infarction^17,18^. *ICMT* encodes a protein-*S*-isoprenylcysteine *O*-methyltransferase that also localizes to the ER and is responsible for cell membrane-targeting of selective proteins^19^. *RNF7* encodes an essential subunit of SKP1-cullin/CDC53-F box protein ubiquitin ligases^20^ and TCEB1 protein is a subunit of the transcription factor B responsible for transcription elongation^21^. Both MFF and OXA1L are mitochondrial proteins localized to outer and inner mitochondria membranes, respectively, and are essential for mitochondria fission and assembly of respiratory complex I^22,23^.

**Fig. 2:**
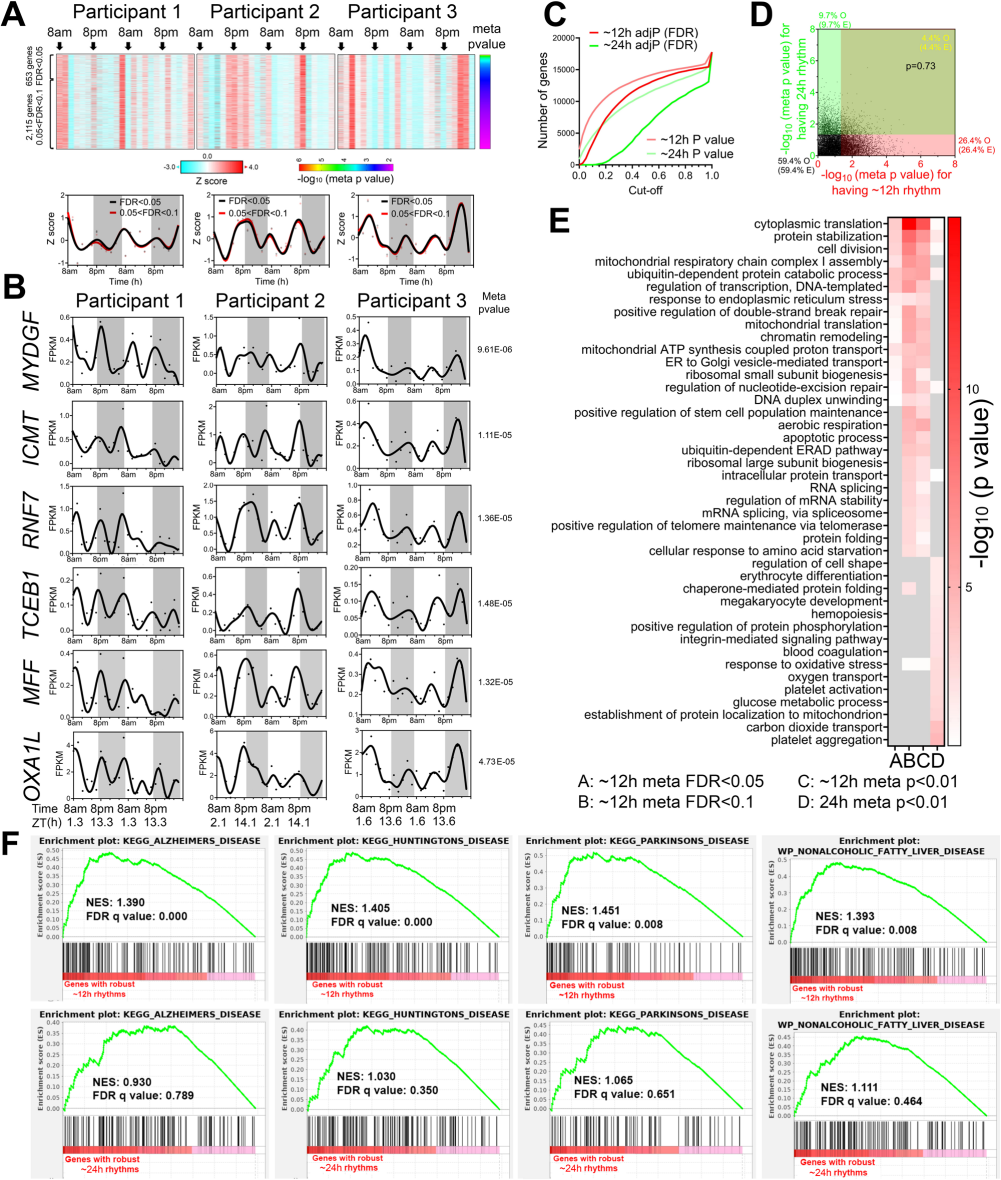
~12-h gene expression rhythms are enriched in mRNA and protein homeostasis pathways. **A** Heatmap and quantification of ~12-h genes from all three participants with meta adj-P (FDR) values less than 0.05 or between 0.05 and 0.1. **B** Raw temporal expression (dot) profile and spline fit (solid line) of six ~12-h genes with meta *p* values also shown. **C** Cumulative distribution of the number of ~24-h and ~12-h genes with different meta p and meta adj-*P* cut-offs. The ~24-h data set consisted of a 4 h sampling interval so that the period to sampling interval was matched to the ~12-h data set. **D** Scatter plot comparing log-transformed meta *p* values for each gene exhibiting ~24-h versus ~12-h rhythms. Both observed and predicted percentage of genes (under the null hypothesis that ~12-h and ~24-h genes are independently regulated) with meta *p* value smaller or larger than 0.05 are further shown. *P* value of 0.73 is calculated by Chi-square test. **E** GO analysis of ~24-h and ~12-h genes with different statistical cut-offs. **F** GSEA showing enrichment scores of different gene sets for ~12-h (top) and ~24-h (bottom) genes.

One important question regarding ultradian rhythmicity is whether it reflects “real’ rhythms with biological significance, or simply mathematical artefact. This is particularly relevant for rhythms that cycle at harmonic frequencies of the 24-h period, as non-sinusoidal circadian waveforms (seesaw-like or square-shaped waveforms) often have superimposed ~12- and ~8-h harmonic frequencies detected by spectral analytical methods such as a Fourier transformation, eigenvalue/pencil, or wavelet analyses^13,24–26^. We took several approaches to address this question. First, 12-h transcriptional oscillations did not cycle at the exact second harmonic of the dominant 22–26-h period in any of the three participants: the first harmonic periods were 22, 18 and 20-h in the three individuals, and 20-h for the meta-analysis (Fig. 1B). Second, if ~12-h oscillations are indeed harmonics of the 22–26-h rhythm, then genes exhibiting both 22–26-h and 10–14-h ultradian rhythmicity should be detected at a higher frequency than expected by chance. To test this, we generated a second set of 24-h gene set using a 4-h interval subset of the original dataset (8am, 12am, 4 pm, etc.) to achieve an equivalent number of data points per period—i.e. equivalent sensitivity—and a meta-p cut-off of 0.05. Importantly, the new 24-h subset showed a strong positive correlation with meta-*p* values obtained with the original full 2-h interval dataset (Fig. S2A). After this adjustment to match sampling interval to period ratios, 10–14-h genes were more prevalent than 24-h genes (Fig. 2C). Moreover, the expected and observed percentages of genes having 24-h and/or 10–14-h rhythms were nearly identical (*p* = 0.73 by Chi-square test), indicating independent detection of these two different rhythms (Fig. 2D). Third, we performed Gene Ontology (GO) and GSEA to compare biological pathways enriched in 22–26-h and 10–14-h genes. We reasoned that if 10–14-h oscillations are mathematical harmonics of the 22–26-h rhythm, then these two groups of genes should share enriched biological pathways. Both analyses revealed largely distinct pathways associated with genes with 22–26-h and 10–14-h rhythms (Fig. 2E and S2B). While the former were enriched in platelet activation, blood coagulation and cell adhesion as previously demonstrated (Fig. 2E and S2B), the latter was associated with fundamental biological processes of mRNA (such as spliceosome and RNA splicing), protein metabolism (including ubiquitin-mediated proteolysis, response to ER stress, protein folding and protein transport in the ER and Golgi), and mitochondria complex chain assembly (Fig. 2E and S2B). To study the potential disease relevance of human 10–14-h genes, we further performed GSEA with disease gene sets. Consistent with the known causal roles of dysregulated proteostasis in proteinopathies^27,28^, and impaired mitochondrial metabolism in metabolic syndromes^29^, 10–14-h genes were strongly enriched in gene sets of neurogenerative disease and nonalcoholic fatty liver disease (Fig. 2F). Enrichment of protein metabolism and mitochondrial respiration genes is also in alignment with ~12-h gene signatures in human dorsolateral prefrontal cortex^30^. Collectively, these analyses support the identification of ~12-h biological rhythms; however, these data do not establish the regulatory mechanism(s) of ~12-h rhythms, which could include cell-autonomous oscillators, homeostatic responses to environmental stimuli, or an “hour-glass“ timing mechanism^31^.

We next performed pathway analyses of the 10–14-h gene sets for each individual participant. We either analyzed the data as a continuous 48-h time series with a single data point at each time point (RAIN conti) or as a 24-h time series where data points collected at the same time on two consecutive days were treated as biological replicates (RAIN dupli) (Figs. S3 and 4, and Table S2). The RAIN duplicate approach revealed larger 10–14-h gene programs: 3462 genes (*p* value < 0.05, with FDR < 0.224), 7060 genes (*p* value < 0.05, with FDR < 0.119) and 4807 genes (p value < 0.05, with FDR < 0.166) for the three participants (Figs. S4A). As expected, 10–14-h genes common to all three individuals also tended to have small meta-*p* values (Fig. S3E and Fig. S4E). Pathways related to mRNA and protein metabolism emerged as significantly enriched for 10–14-h rhythm genes in each participant regardless of the inputs or thresholds for RAIN analysis (Figs. S3G and S4C, E).

To determine robustness to different analytic methods, we also performed spectrum analysis with the eigenvalue/pencil method^11,13,14,32–34^, which unlike statistical methods such as JTK_CYCLE and RAIN does not require pre-assignment of period range, enabling unbiased identification of multiple oscillations for any given gene^11,13,14,32–34^. Eigenvalue/pencil analyses also revealed ~24-h and ~12-h oscillations in all three individuals (Figs. S5–7, and Table S3). ~12-h gene programs were distinct from ~24-h gene sets in all three individuals and enriched in mRNA and protein metabolism pathways (Figs. S5J, S6J and S7J). Taken together, orthogonal analytical methods and statistical thresholds suggested ~12-h rhythms of gene expression implicated in mRNA and protein metabolism in human white blood cells.

To infer gene regulatory networks governing ~12-h rhythms, we performed LISA^35^ and motif analysis on the top 500 10–14-h genes uncovered by the RAIN method with the lowest meta-*p* values. We cross-referenced enriched motifs with transcription factor genes that exhibited 10–14-h rhythms (with meta-adjP<0.1), identifying XBP1, CREBZF, GABPB1/2, NFYB, GMEB1, KLF16 as top candidates that were preferentially enriched with 10–14-h genes compared to 22–26-h genes (Fig. 3A–D). XBP1, CREBZF and CREB1 belong to the Basic Leucine Zipper (bZIP) transcription factor family and are established transcription regulators of the unfolded protein response (UPR) and adaptive stress response^36–40^. GABPB1/B2 are ETS family transcription factors involved in mitochondria biogenesis and oxidative phosphorylation^41,42^. NFYB encodes one of the three subunits of nuclear transcription factor Y (the other being NFYA and NFYC) and its DNA binding motif was recently shown to co-occur with XBP1 in mouse type-2 T helper cells^43^. Several of these transcription factors have previously been implicated in regulation of ~12-h rhythms in mice. For instance, it was previously proposed that ~12-h rhythms are generally established by a tripartite network comprising ETS, bZIP and NFY transcription factors, whereas KLF transcription factors may control ~12-h rhythms in a more tissue specific manner^44^. Enriched GMEB DNA binding motifs were also observed in the promoters of hepatic ~12-h rhythms in mice^32^. We next incorporated a recently published XBP1 ChIPmentation dataset from mouse T helper cells into our analysis^43^. We found that XBP1 target genes—those genes directly bound by XBP1 and whose expression is reduced in the absence of XBP1—were significantly enriched in the putative 10–14-h human gene set, but not in the 22–26-h gene set (Fig. S8A–C). These results suggest XBP1, GABP and KLF transcription factor family members as candidate transcriptional regulators of ~12-h rhythms, with XBP1 a strong candidate given its previously identified role as a major regulator of ~12-h transcriptional oscillations in the murine liver^14^.

**Fig. 3:**
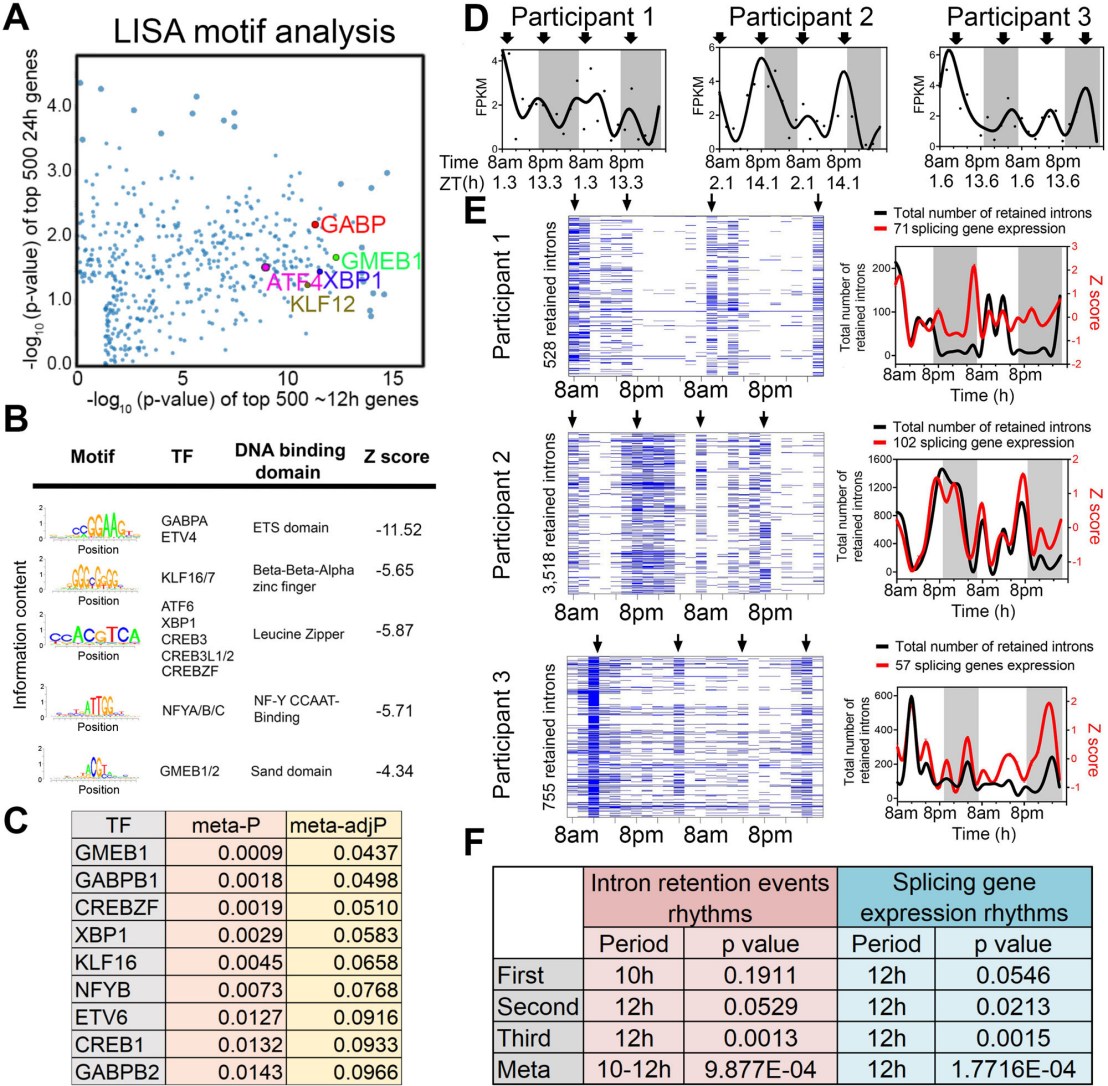
Regulatory and functional dissection of human ~ 12-h rhythms. **A** Scatter plot demonstrating the log normalized *p* values of motifs identified for top 500 (ranked by meta *p* values) ~12-h (x axis) versus the ~24-h genes using the LISA program, with selective TFs highlighted. **B** Top motifs enriched at the promoters of ~top 500 12-h genes using the SeqPos motif tool in Galaxy/Cistrome. **C** A table listing the TFs whose motifs are enriched in the promoters of ~12-h genes and whose gene expression also exhibit ~12-h rhythms with meta adj-P less than 0.1. **D** Temporal expression of *XBP1* in all three individuals. Criterions for IR are set as *T* > = 20, *J* > = 1, FPKM > = 2 and NE score > =0.9. Heatmap (left) and quantification (right) of temporal intron retention events, superimposed with the Z score normalized temporal expression of splicing genes exhibiting ~12-h rhythms (**E**). Statistics for IR and spicing gene expression ~12-h rhythms detection by RAIN (**F**).

Given representation of RNA metabolism and mRNA splicing pathways in ~12-h transcriptional rhythms, we tested whether such oscillations would translate into a downstream functional effect in the form of alterations in mRNA splicing. We interrogated the RNA-seq data for evidence of rhythmicity in intron retention (IR) events, predicting that if rhythmicity of RNA splicing genes was functionally relevant, it would temporally correlate with global IR. We applied a recently published algorithm iREAD^45^. Using two different criteria to define retained introns, we identified 10–12-h rhythms of global IR events in all three participants (with meta-*p* < 0.003) (Fig. 3E, F and S9A–D). IR rhythms were synchronized to the expression of mRNA splicing genes (Fig. 3E, F and S9A–D). We next performed GO analysis on the set of transcripts in which we detected retained introns. While GO terms associated with intron retention genes were largely consistent at the morning and evening peaks, we also observed differential enrichment in a subset. The morning intron retention gene sets were enriched in immune functions, especially those involving the display of intracellular peptide fragments to cytotoxic T cells via MHC class 1 complexes (e.g., HLA-A/C/E and B2M), whereas the evening intron retention gene sets exhibited greater heterogeneity across the three participants (Figs. S10, 11). This data suggests synchronization of mRNA splicing gene programs to splicing functionality, consistent with a potential role for ~12-h rhythms in RNA metabolism.

~12-h rhythms in coastal and estuarine animals suggest an ancient evolutionary origin^2,44,46–50^ (Fig. 4A). To test for potential homology between human ~12-h rhythms and circatidal genes in marine animals, we compared the most robust 10–14-h human genes (meta-adjP less than 0.10) with the circatidal gene program uncovered in *Aiptasia diaphana*^48^, a sea anemone species which shares a eumetazoan ancestor with *Homo sapiens* ~ 700 million years ago in the Cryogenian period^51^. In line with our human data, the most enriched biological pathways amongst *A. diaphana* circatidal genes are related to mRNA processing and protein metabolism, distinct from those enriched in the circadian genes, which included pathways of carbohydrate metabolism and detoxification^48^ (Fig. S12A). We found 404 ~ 12-h genes common to both species, a number significantly higher than the expected value of 263 if there was no evolutionary conservation (*p* = 4.6e-23 by Chi-square test) (Fig. 4B). This subset was also enriched in mRNA splicing and proteostasis pathways (Fig. 4C, D). We further examined potential conservation with two additional human 10–14-h gene sets: 653 gene set with meta-adjP values less than 0.05 (Fig. 1C), or the 851 gene set shared in all three individuals depicted in Fig. S4B (Fig. S12B, C). Protein processing in the ER was the most consistently enriched GO term using the different selection criteria for ~12-h genes (Fig. S12B, C). We also found evidence of conserved ~24-h gene expression between human (with meta-adjP value less than 0.10) and *A. diaphana* (*p* = 0.049 by Chi-square test), with the 102 common genes enriched in carbohydrate metabolism and detoxification pathways (Fig. S12D–F).

**Fig. 4:**
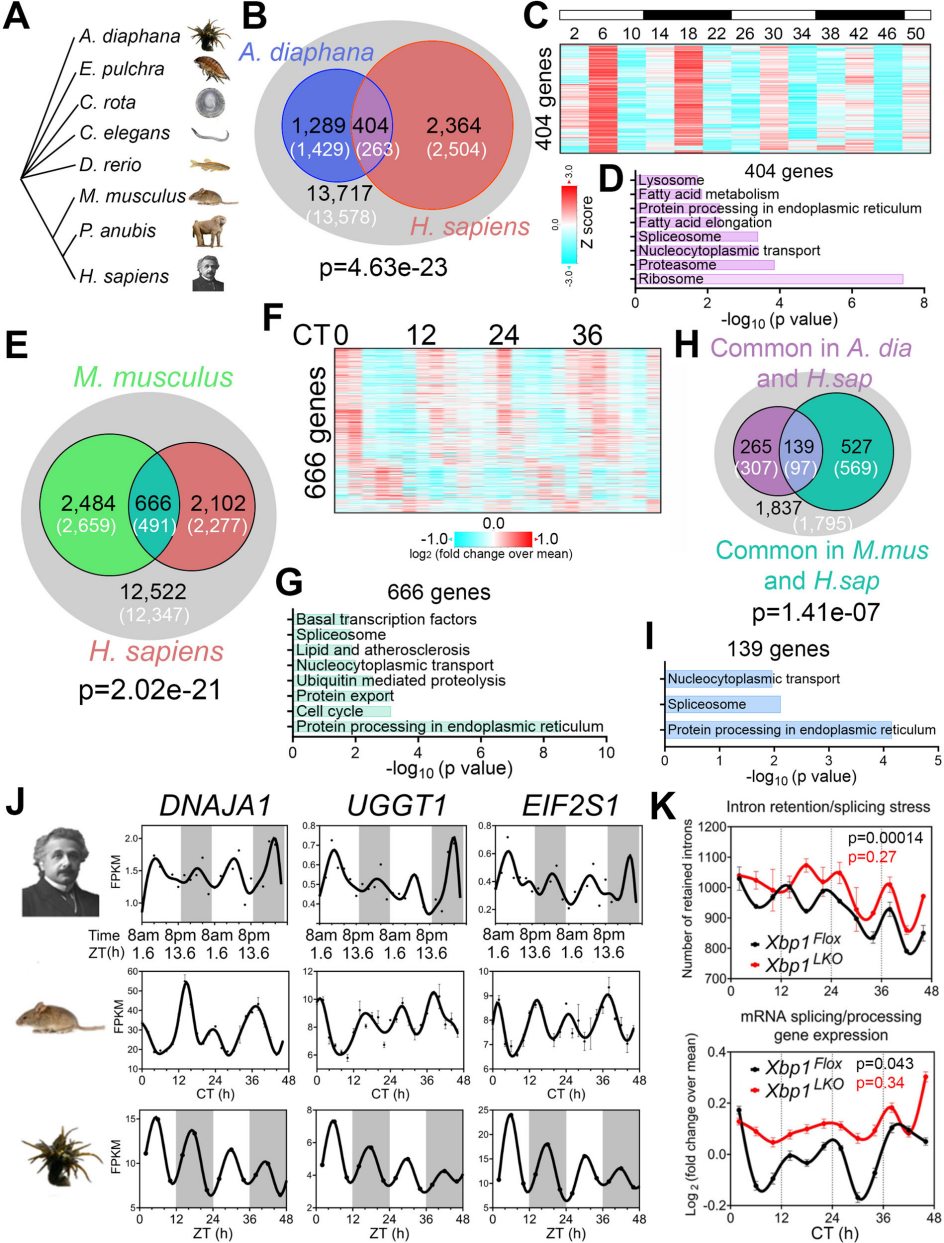
Evolutionary conservation of ~ 12-h gene programs. **A** Phylogenetic tree of select species for which ~12-h rhythms of gene expression have been demonstrated. *A. diaphana* is the most distant from *H. sapiens*. **B** Venn diagram comparing distinct and shared ~12-h genes in human (meta adj-*P* < 0.1) and *A. diaphana* (reported in^48^). Only genes expressed in human white blood cells (denoted by the grey circle) are included in the analysis. Both observed and predicted number of genes (under the null hypothesis that ~12-h genes are not evolutionarily conserved and thus independently detected in these two species) are further shown. *P* value of 4.6e-23 is calculated by Chi-square test. Heatmap of temporal expression (Z score normalized) of 404 circatidal genes in *A. diaphana* (**C**) and GO analysis of the top enriched pathways (**D**). **E** Venn diagram comparing distinct and shared ~12-h genes in human (meta adj-P < 0.1) and mouse liver (reported in ref. ^14^). Only genes that are expressed in human white blood cells (denoted by the grey circle) are included in the analysis. Both observed and predicted number of genes (under the null hypothesis that ~12-h genes are not evolutionarily conserved and thus independently detected in these two species) are further shown. *P* value of 2.0e-21 is calculated by Chi-square test. Heatmap of temporal expression (log 2 normalized) of 666 ~ 12-h genes in mouse liver (**F**) and GO analysis of the top enriched pathways (**G**). **H** Venn diagram further comparing the overlap of commonly identified 404 ~ 12-h genes in human and A. diaphana, and 666 ~ 12-h genes in human and mouse. Both observed and predicted number of genes (under the null hypothesis that ~12-h genes are not evolutionarily conserved and thus independently detected in all three species) are further shown. *P* value of 1.4e-7 is calculated by Chi-square test. **I** GO analysis of the top enriched pathways of 139 ~ 12-h genes commonly identified in all three species. **J** Representative temporal expression of evolutionarily conserved ~12-h genes in human, mouse, and *A. diaphana*. **K** ~ 12-h rhythms of intron retention events (top) and RNA splicing genes expression (bottom) are attenuated by liver-specific loss of function of XBP1 (*Xbp1*^*LKO*^: red), relative to control (*Xbp1*^*Flox*^: black) in mice. *P* values calculated by RAIN for ~12-h rhythms.

If the ~12-h gene program is conserved from coastal invertebrates to humans, we reasoned that lower mammals like mice should also exhibit oscillations in similar gene programs. We re-examined a previously published temporal hepatic RNA-seq data set in mouse liver and found 666 ~ 12-h genes common to both human and mouse (*p* = 2.02e-21 by Chi-square test), again highly enriched in mRNA splicing and protein processing pathways (Fig. 4E–G). We identified 139 ~ 12-h genes common to all three species: human, mouse, and *A. diaphana*, which exceeded the 97 expected by chance (*p* = 1.41e-7 by Chi-square test) (Fig. 4H). These 139 genes are enriched in protein processing in ER, spliceosome, and nucleocytoplasmic transport, the three pivotal steps in central dogma information flow. Specific examples include DNAJA1, a heat shock protein 70 cochaperones^52^, UGGT1 that serves as the predominant ER glycoprotein quality control sensor^53^ and *EIF2S1*, encoding the alpha subunit of the translation initiation factor eIF2 protein complex^54^ (Fig. 4I, J). These results are congruent with our previous work in murine liver in identifying a regulatory function for ~12-h oscillator in central dogma information flow^14^.

Finally, we tested for synchronization between the ~12-h rhythms and RNA splicing in mice by performing intron retention analysis of our previously published temporal RNA-seq data from mouse liver^14^. Like the human data, we observed alignment between ~12-h rhythms in splicing gene expression and global intron retention events peaking at CT2 and CT14 (Fig. 4K). Liver-specific genetic ablation of XBP1 (a transcriptional regulator of murine hepatic 12-h oscillations) weakened ~12-h rhythms of splicing gene expression and intron retention rhythms, leading to constant intron retention across the day (Fig. 4K). These collective data support evolutionary conservation of a ~ 12-h gene program in humans related to RNA and protein metabolism.

In this study, we discovered ~12-h gene programs related to fundamental processes of mRNA and protein metabolism in humans. Remarkably, pathways and specific gene orthologs exhibiting ~12-h circatidal rhythms in marine animals^55^ were also detected in humans. Our discovery of one such ~12-h pathway, ‘mRNA splicing,’ provided an opportunity to establish functional significance through identification of corresponding ~12-h rhythms in global intron retention events. While our study provides evidence of ~12-h rhythms of gene expression in humans, it does not establish causality, nor does it fully address their relationship with the circadian clock. At least three mutually non-exclusive mechanisms have been proposed to explain the origin and regulatory mechanisms of ~12-h rhythms in mice, namely that they are not cell-autonomous and controlled by a combination of the circadian clock and environmental cues^26,56,57^, that they are regulated by two anti-phase circadian transcriptional factors in a cell-autonomous manner^58^, or that they are established by a cell-autonomous ~12-h oscillator^13,14,32–34,59^. Future studies will be required to test whether candidate 12-h regulatory factors are causal drivers of ~12-h gene expression rhythms and any direct relationship with the circadian clock in humans.

How ~12-h rhythms in mRNA and protein metabolism confer an evolutionary advantage to non-coastal terrestrial mammals is an open question. One possibility is that ~12-h rhythms have been co-opted by terrestrial mammals as an adaptive response to ~12-h metabolic stress cycles that peak at critical transitions^10^. The absence of energy intake during sleep crescendos to a peak of energy shortage at dawn. Conversely, at dusk, a reduction in active energy expenditure coupled with cumulative energy intake throughout the day, drives peak positive energy balance. Both ends of the energy balance spectrum are metabolically stressful and activate XBP1s, thereby triggering the UPR^44,60,61^. Therefore, we speculate that mammalian ~12-h rhythms provide an anticipatory advantage to address twice daily metabolic stress.

## Methods

### Human participants and study protocol

The study protocol was approved by the University of Pittsburgh Institutional Review Board (Study 20020034; approval date: 6/4/2020) and written consent was obtained from all study participants. We studied 3 healthy male participants, who were recruited through online advertisements. Inclusion criteria consisted of individuals 18–35 years of age with a self-reported regular nighttime sleep schedule and a body mass index between 18.5–24.9 kg/m^2^. Volunteers were excluded if they admitted to nighttime shift work or other regular nighttime sleep-disrupting activities, if they had any chronic medical conditions, took any medications or recreational drugs, or used tobacco products. Potential volunteers presented for a screening visit, inclusive of measurement of body weight, height, BMI, and laboratory studies, including a comprehensive metabolic panel (electrolytes, kidney function and liver function tests), complete blood count, 25-OH vitamin D, and thyroid stimulating hormone level, to screen for potential subclinical chronic diseases. We excluded participants with low hemoglobin/hematocrit, abnormal thyroid function and individuals with 25-OH vitamin D < 20 ng/mL.

Qualifying study participants maintained a food diary, which was used to estimate their daily caloric intake and subsequently presented to the University of Pittsburgh Medical Center (UPMC) Clinical Translational Research Center (CTRC) for a 3-day inpatient visit. On the morning of admission, participants selected items from a food menu designed to match their standard daily caloric intake with a uniform macronutrient composition of 55% carbohydrates, 25% fat, 20% protein per day. No interventions were performed during the first 24-h period of acclimatization to the hospital. Each study participant was housed in the same room in the CTRC, which contained a window to the outside. The participants were not entrained to any specific environmental cues during the study. They were encouraged to maintain their usual sleep-wake cycles; however, they maintained control over the lighting in the room. They were free to ambulate in the hallway of the CTRC; however, they did not leave the unit during the 3-day stay. The three meals were delivered at roughly the same time each day based on the standard CTRC schedule (8 A.M., 12 P.M., 6 P.M.); however, there were no rules dictating the duration of their meals. These measures were implemented to mimic free-living conditions. On the morning of the second day, an intravenous (IV) line was placed to commence blood collections at 8 A.M. and then every 2 h for 48 h (total 24 samples). Nighttime blood collection was performed through a long IV line from outside the room so that the participant would not be exposed to light or disturbed during blood collection. Blood was immediately processed in the Center for Human Integrative Physiology, two floors above the UPMC CTRC by a rotating study team, all of whom were trained in the processing procedures for this study. Blood was centrifuged (1900 RCF x 10 min) and the buffy coats were collected and immediately snapped frozen in liquid nitrogen for storage at −80C.

### RNA-Seq and data analysis

RNA was isolated from peripheral blood buffy coat samples on the automated Chemagic 360 (Perkin Elmer) instrument according to the manufacturer’s instructions. Extracted RNA was quantitated by Qubit™ RNA BR Assay Kit (Thermo Fisher Scientific) followed by RNA quality check using Fragment Analyzer (Agilent). For each sample, RNA libraries were prepared from 100 ng RNA using the KAPA RNA HyperPrep Kit with RiboErase (Kapa Biosystems) according to manufacturer’s protocol, followed by quality check using Fragment Analyzer (Agilent) and quantification by qPCR (Kapa qPCR quantification kit, Kapa biosystem) on the LightCycler 480 (Roche). The libraries were normalized and pooled, and then sequenced using NovaSeq6000 platform (Illumina) to an average of 40 M 101 bp paired end reads per sample. Low-quality reads and adapter sequences were trimmed from the raw sequencing data with Trimmomatic^62^. The remaining reads were then aligned to human reference genome hg38 with STAR aligner^63^. Gene counts were quantified with the STAR-quantMode GeneCounts function. Fragments per kilobase of transcript per million mapped fragments (FPKM) were quantified with Cufflinks^64^.

### Identification of the oscillating transcriptome

Averaged FPKM values at each time were used for cycling transcripts identification. Lowly expressed transcripts were removed by calculating the background expression in each participant using the average expression of a panel of 62 genes known not to be expressed in peripheral blood cells (Table S2). Temporal transcriptomes were subjected to linear detrend prior to identification of oscillations by either the eigenvalue/pencil or RAIN methods. For the eigenvalue/pencil method^11,13^, a maximum of four oscillations were identified for each gene. Criterion for ~24-h genes were: period between 20 h to 25 h for first and second participants and 24 h to 30 h for the third participant, decay rate between 0.8 and 1.2; for ~12-h genes: period between 9.6 h to 13.6 h for the second and third participants and 10 h to 14 h for the first participant, decay rate between 0.8 and 1.2; for ~8 h genes: period between 6 h to 8 h for the first participant and 7 h to 9 h for the second participant, decay rate between 0.8 and 1.2; for ~16 h genes; period between 14 h to 18 h for the third participant. The relative amplitude was calculated by dividing the amplitude by the mean expression value for each gene. To determine FDR, we used a permutation-based method that randomly shuffles the time label of gene expression data and subjected each permutation dataset to the eigenvalue/pencil method applied with the same criterion^65^. These permutation tests were run 5000 times, and FDR was estimated by taking the ratio between the mean number of rhythmic profiles identified in the permutated samples (false positives) and the number of rhythmic profiles identified in the original data. Analyses were performed in MatlabR2017A. RAIN analysis was performed as previously described in Bioconductor (3.4) (http://www.bioconductor.org/packages/release/bioc/html/rain.html) with either 48-h continuous data or 24-h data with biological duplicates as input^12^. Genes exhibiting a period range between 10-h and 14-h and a period range between 22-h and 26-h are considered as ~12-h and ~24-h genes, respectively. FDR was calculated using the Benjamini-Hochberg procedure. Heat maps were generated with Gene Cluster 3.0 and TreeView 3.0 alpha 3.0 using Z score normalized values.

For meta-analysis, we used Fisher’s method, which combines extreme value probabilities from each test, commonly known as “*p* values”, into one test statistic (X2) using the formula

1 $${X}_{2k}^{2}=-2{\sum \limits_{i=1}^{k}}{\mathrm{ln}}\,{p}_{i}$$

where pi is the *p* value for the ith hypothesis test. For meta-analysis of ~12-h (10–14-h) and ~24-h genes (22–26-h), the smallest *p* value for each gene within these period ranges was used for each individual. For example, *XBP1* was found to oscillate with periods of 12-h (*p* = 0.068), 12-h (*p* = 0.014) and 10-h (*p* = 0.049) in participant 1, 2, and 3, respectively, and the meta *p* values for being a common ~12-h gene (cycling with a period between 10-h and 14-h) were calculated by “merging” the three respective *p* values (meta *p* = 0.0029). The same procedure was also performed for genes cycling with a period between 22-h and 26-h. This meta-analysis is feasible because the statistical test for each gene in each individual shares the same null hypothesis: absence of rhythms.

For RAIN analysis on temporal IR events and splicing gene expression, raw data was subjected to polynomial detrend (*n* = 2) before RAIN analysis.

### Defining oscillating genes

The eigenvalue method can detect multiple superimposed oscillations. Therefore, we defined a ~ 24-h gene as one that exhibited a ~ 24-h rhythm, regardless of its amplitude relative to other superimposed oscillations. Similar criteria were applied to other oscillations. As such, a gene can meet criteria for both a ~ 24-h and ~12-h gene. By comparison, we define a dominant ~24-h gene as one in which the superimposed ~24-h rhythm has the largest amplitude among all oscillations. With this definition, dominant ~24-h and dominant ~12-h genes are mutually exclusive.

### Intron retention detection

Intron retention events were detected by tool iREAD^45^. Intron retention events are selected either with default settings *T* > = 20, *J* > = 1, FPKM > = 2 and NE score > =0.9 or more stringent settings where *T* > = 20, *J* > = 1, FPKM > = 3 and NE score > =0.9.

### Gene ontology analysis

DAVID (Version 2021)^66^ (https://david.ncifcrf.gov) was used to perform Gene Ontology analyses. Briefly, gene names were first converted to DAVID-recognizable IDs using Gene Accession Conversion Tool. The updated gene list was then subject to GO analysis using all Homo Sapiens as background and with Functional Annotation Chart function. GO_BP_DIRECT, KEGG_PATHWAY or UP_KW_BIOLOGICAL_PROCESS were used as GO categories. Only GO terms with a *p* value less than 0.05 were included for further analysis.

### Motif analysis

Motif analysis was performed with the SeqPos motif tool (version 0.590) embedded in Galaxy Cistrome using all motifs within the homo sapiens reference genome hg19 as background. LISA analysis was performed using web tool (http://lisa.cistrome.org/).

### Supplementary information

Supplementary Materials

Table S1 RPKM values of gene expression in three humans

Table S2 RAIN analysis of ~12h genes

Table S3 Eigenvalue results for three humans

## Data Availability

All raw and processed sequencing data generated in this study have been submitted to the NCBI Gene Expression Omnibus (GEO; http://www.ncbi.nlm.nih.gov/geo/) under accession numbers GSE220120.
