# Supplementary material for: Evidence for ~12-h ultradian gene programs in humans
Source: NPJ Biol Timing Sleep. 2024 Aug 9;1:4. doi: 10.1038/s44323-024-00005-1 (PMC11325440; doi:10.1038/s44323-024-00005-1)
Supplement: Supplementary file 1 — Supplementary Materials [file 44323_2024_5_MOESM1_ESM.pdf]

Supplementary Materials for

Evidence for ~12-hour ultradian gene programs in humans

Bokai Zhu[1,2,3] *, Silvia Liu[2,4], Natalie L. David[1,5,6], William Dion[1], Nandini K Doshi[1,6], Lauren B. Siegel[5], Tânia Amorim[1,5,6], Rosemary E. Andrews[1,6], GV Naveen Kumar[1], Hanwen Li[7], Saad Irfan[1], Tristan Pesaresi[1,6], Ankit X. Sharma[1], Michelle Sun[1], Pouneh K. Fazeli [5,6] and Matthew L. Steinhauser [1,6,8] *

Corresponding author: bzhu@pitt.edu, msteinhauser@pitt.edu

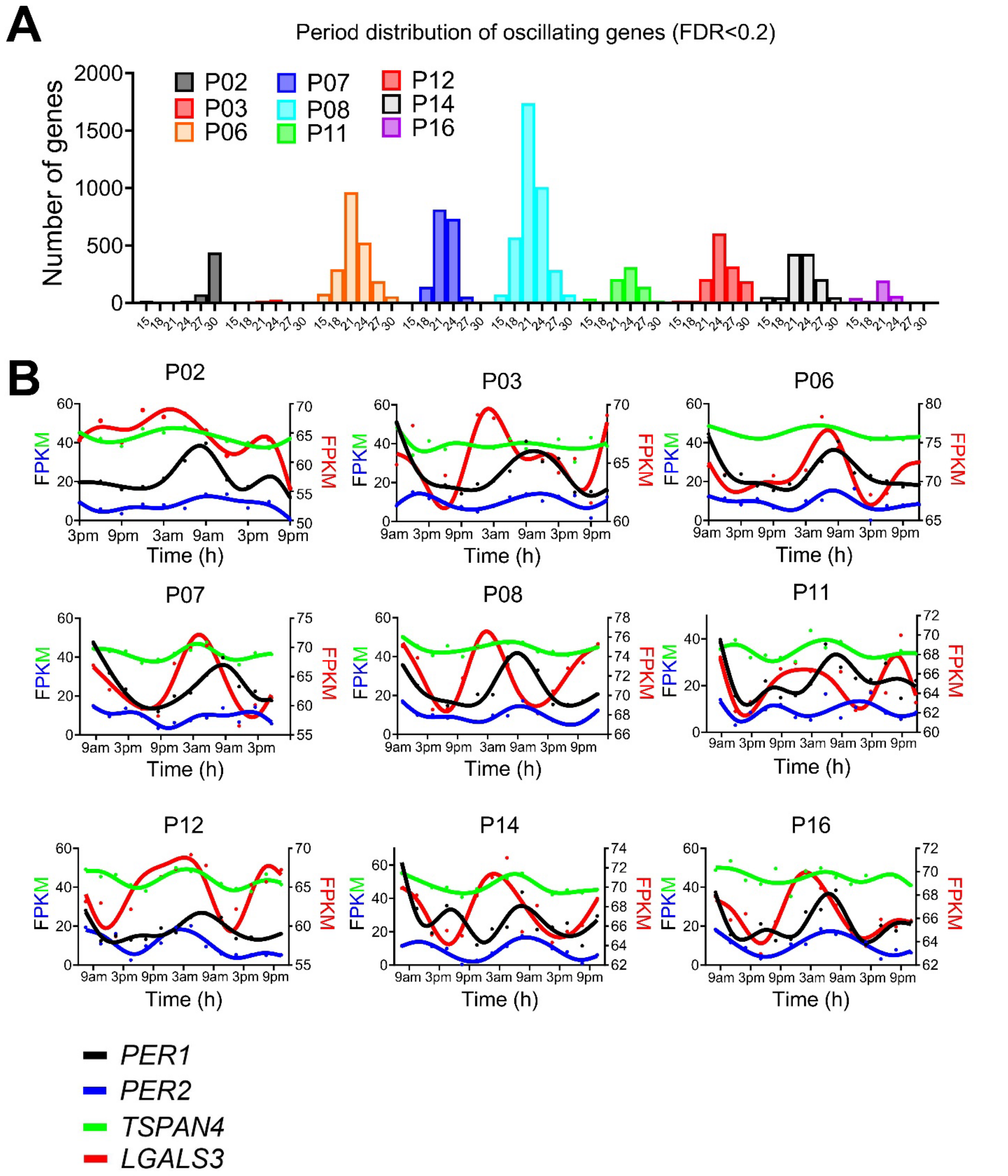

Fig. S1. Inter-individual variability of ~24-hour rhythms of gene expression reported in the Wittenbrink et al JCI study. (**A**) Distribution of periods for gene oscillations between 15 and 30 hours and (**B**) Raw temporal expression profile (dot) and spline fit (solid line) of *PER1* (black), *PER2* (blue), *TSPAN4* (green) and *LGALS3* (red) genes in nine different individuals, reported in [15].

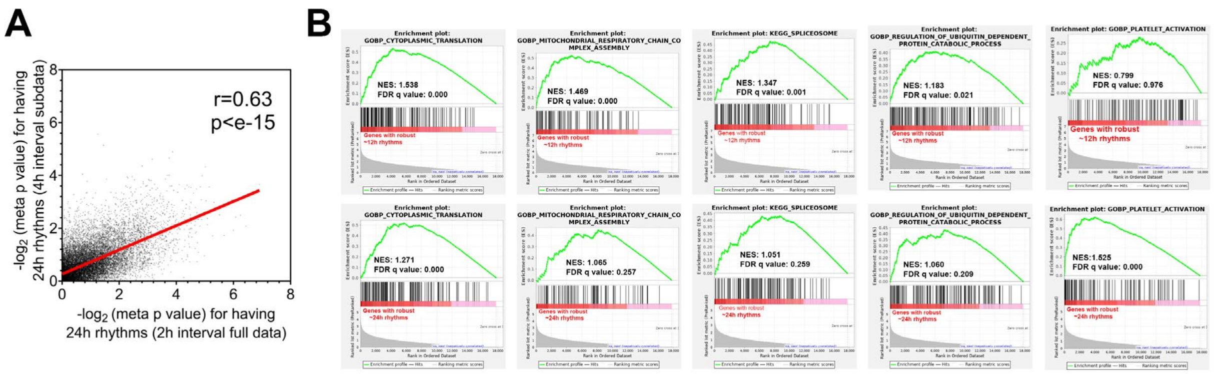

Fig. S2. Genes with ~12h and ~24-hour rhythms are enriched in distinct biological pathways. (**A**) Scatter plot comparing log normalized meta p values for having ~24-hour rhythms for each gene when the full 2h sampling interval dataset (x-axis) or the 4h sampling interval subset (y-axis) was used for the analysis. (**B**) GSEA showing enrichment scores for different gene sets in ~12-hour (top) and ~24-hour (bottom) genes.

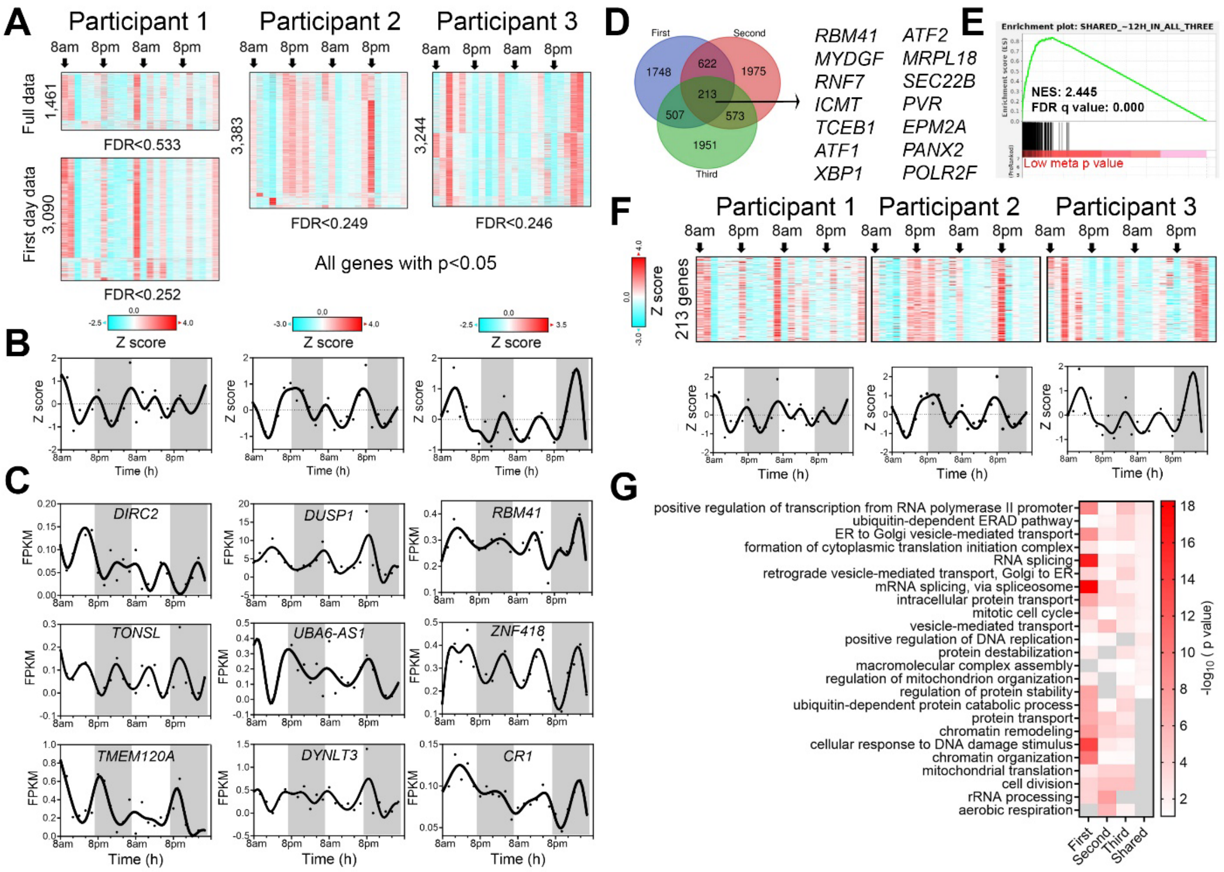

Fig. S3. RAIN analyses of 48-hour temporal transcriptome to detect ~12-hour genes. A single data point was inputted for each sample collected over the 48h protocol. (**A**) Heatmap of ~12-hour genes from all three participants with respective p values smaller than 0.05. The identification of a greater number of ~12-hour genes with lower FDR in the first participant with restriction of inputted data to the first 24 hours indicates the ~12-hour rhythm is dampened in the second day in this participant, consistent with what was found with the eigenvalue/pencil method. (**B**) Quantification of the average expression (Z score normalized) of genes shown in A, with raw data (dots) and spline fit (solid lines) shown. (**C**) Raw temporal expression (dot) profile and spline fit (solid line) of top three ~12-hour genes with the smallest p values in each of the three individuals. (**D**) Venn diagram depicting common and distinct ~12-hour genes for each individual, with selective common genes shown on the right. (**E**) GSEA showing enrichment score of 213 common ~12-hour genes on robust ~12-hour genes ranked by meta p values. (**F**) Heatmap and quantification of 213 common ~12-hour genes uncovered from all three participants. (**G**) GO analysis of all and shared ~12-hour genes in each individual.

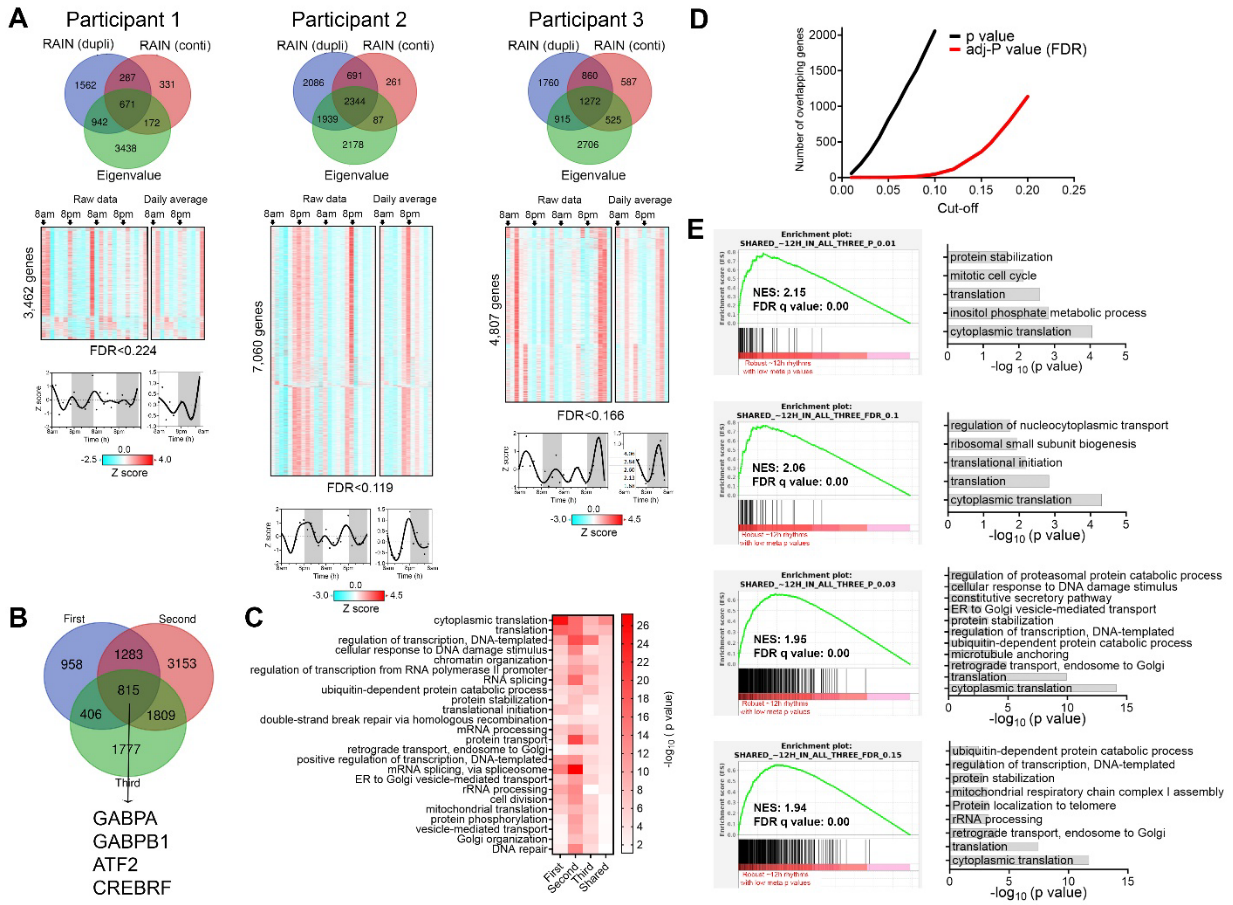

Fig. S4. RAIN analyses of temporal transcriptomes using consecutive 24 hours datasets as biological replicates. (**A**) Venn diagram comparing the ~12-hour programs uncovered by the three methods (top) and heatmap and quantification of ~12-hour genes uncovered by the RAIN dupli method in all three participants (bottom). (**B**) Venn diagram depicting common and distinct ~12-hour genes uncovered in each participant. (**C**) GO analysis of all and shared ~12-hour genes in all three participants. (**D**) Scatter plot comparing the number of overlapping ~12-hour genes between the three individuals against different p values or FDR cut-offs for all individuals. (**E**) GSEA showing enrichment scores for four gene sets of overlapping ~12-hour genes with p<0.01 (56 genes), p<0.03 (358 genes), FDR<0.1 (46 genes) and FDR<0.15 (361 genes) cut-off (left) on ~12-hour genes ranked by meta-p values and GO analysis of these genes (right).

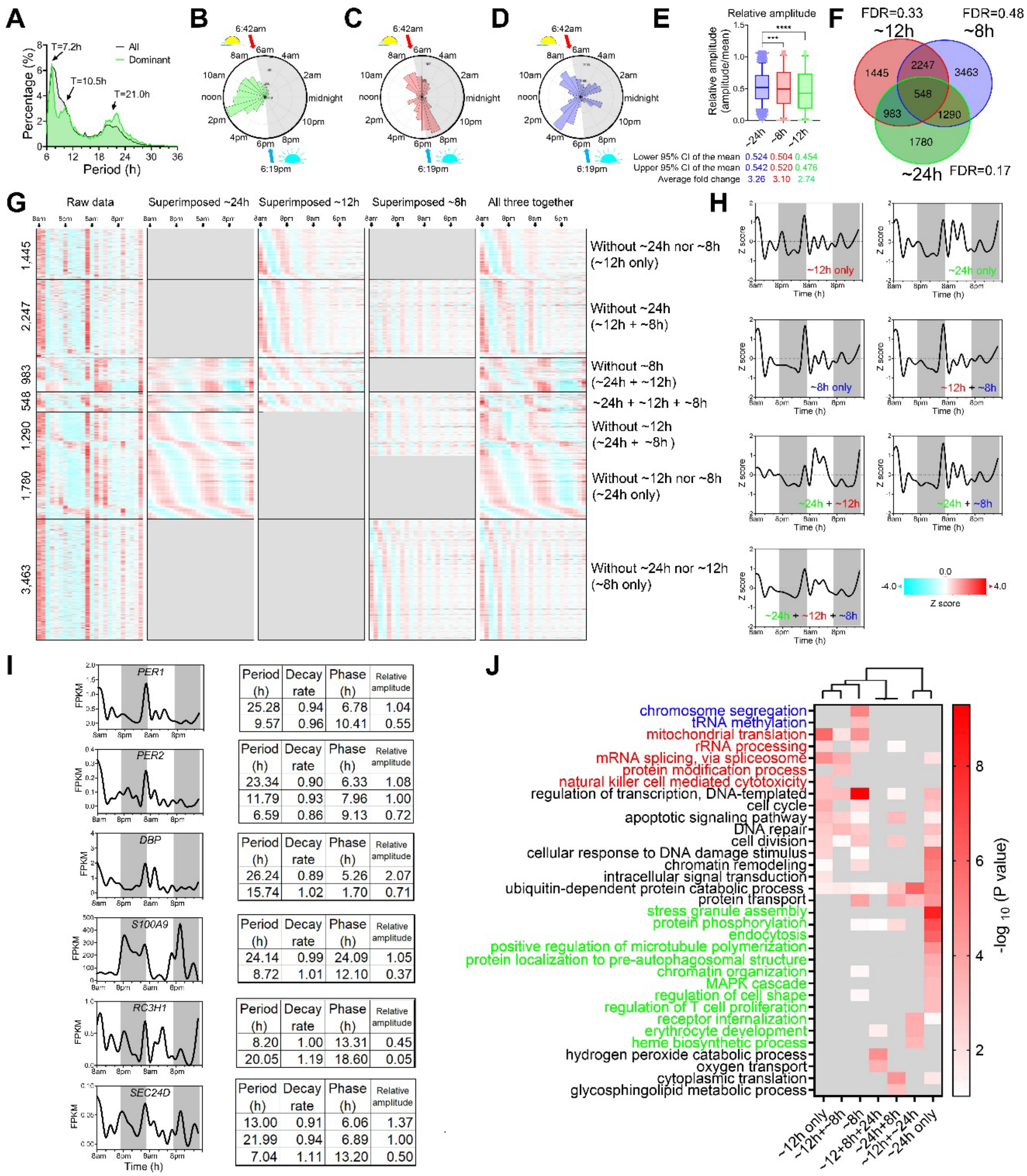

Fig. S5. Transcriptome spectrum of first human participant. (**A**) Period distribution of all and dominant oscillations. (**B-D**) Polar histogram illustrating the phase distribution of ~24-hour (**B**), ~12-hour (**C**) and ~8-hour (**D**) oscillations. (**E**) Relative amplitude (mean-normalized) of ~8-hour, ~12-hour and ~24-hour oscillations. (**F**) Venn diagram showing distinct and shared oscillations for each period. (**G, H**) Heatmap (**G**) and quantification (**H**) of decomposition of raw temporal transcriptome into harmonics cycling at ~8-hour, ~12-hour or ~24-hour periods. (**I**) Representative temporal expression of select genes and eigenvalue/pencil decomposition uncovering all superimposed oscillations for each gene. (**J**) GO analysis of all genes in **G**.

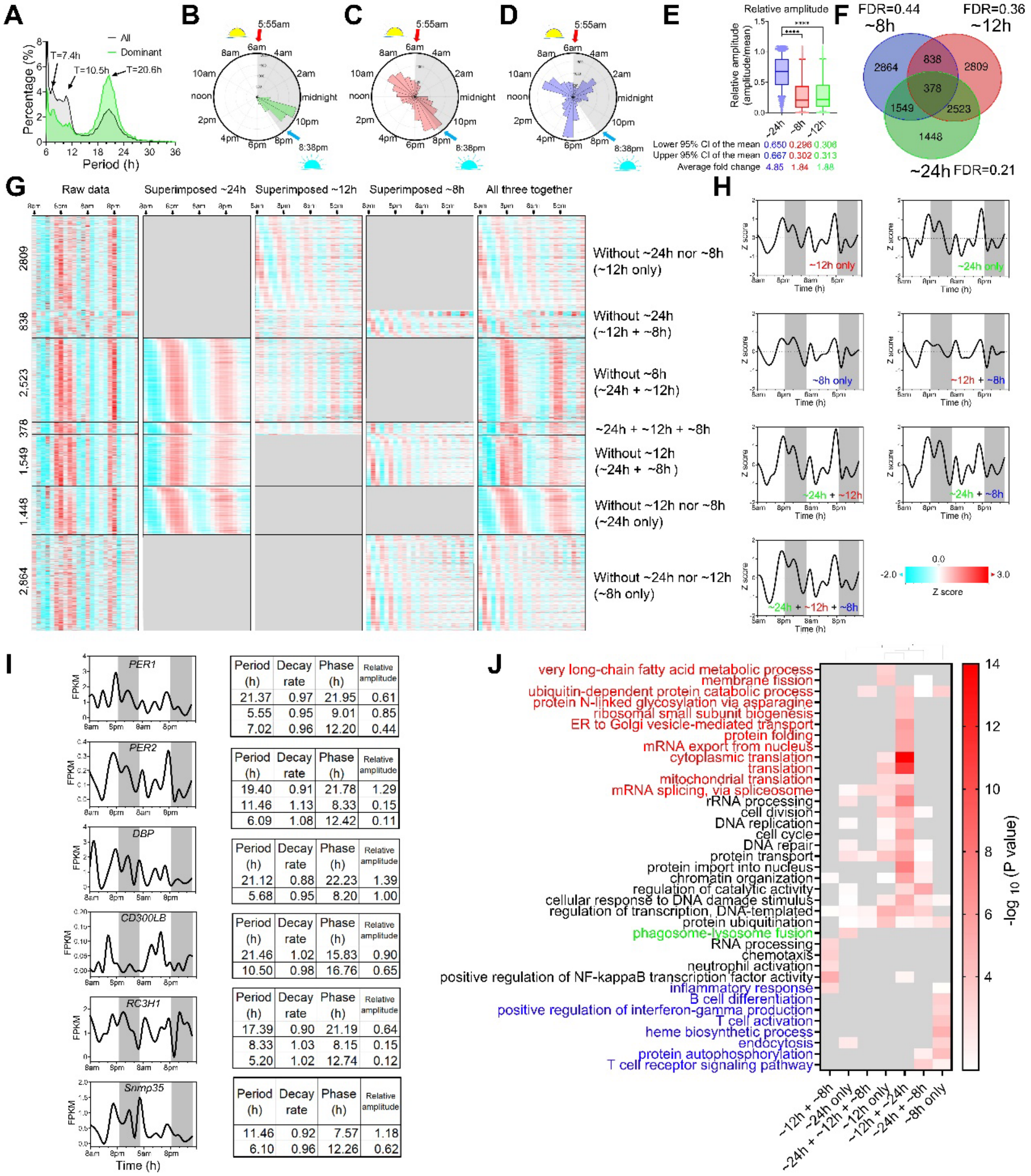

Fig. S6. Transcriptome spectrum of the second human participant. (**A**) Period distribution of all and dominant oscillations. (**B-D**) Polar histogram illustrating the phase distribution of ~24-hour (**B**), ~12-hour (**C**) and ~8-hour (**D**) oscillations. (**E**) Relative amplitude (mean-normalized) of ~8-hour, ~12-hour and ~24-hour oscillations. (**F**) Venn diagram showing distinct and shared oscillations for each period. (**G, H**) Heatmap (**G**) and quantification (**H**) of decomposition of raw temporal transcriptome into harmonics cycling at ~8-hour, ~12-hour or ~24-hour periods. (**I**) Representative temporal expression of selective genes and eigenvalue/pencil decomposition uncovering all superimposed oscillations for each gene. (**J**) GO analysis of all genes in **G**.

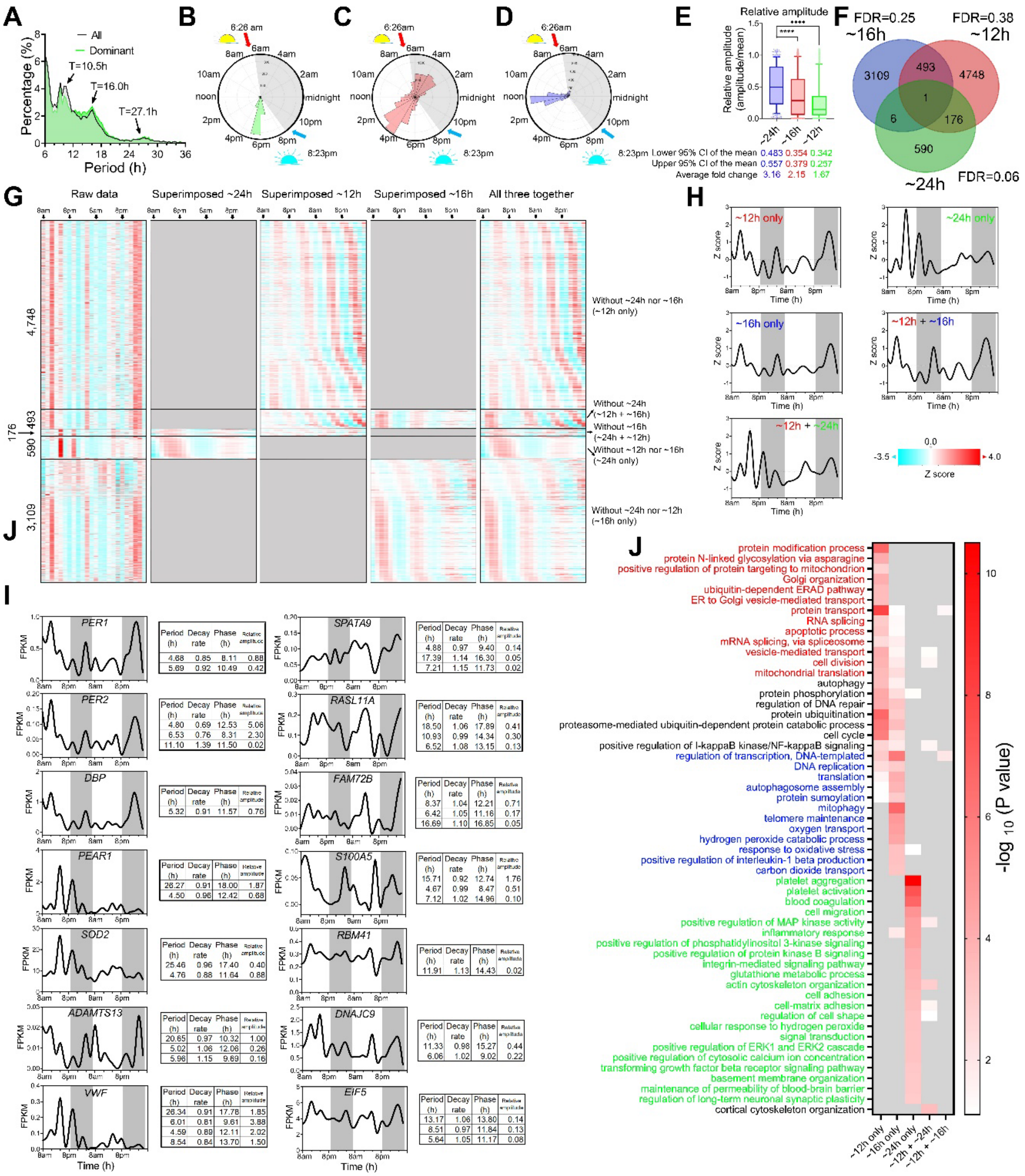

Fig. S7. Transcriptome spectrum of the third human participant. (**A**) Period distribution of all and dominant oscillations. (**B-D**) Polar histogram illustrating the phase distribution of ~24-hour (**B**), ~12-hour (**C**) and ~16-hour (**D**) oscillations. (**E**) Relative amplitude (mean-normalized) of ~16-hour, ~12-hour and ~24-hour oscillations. (**F**) Venn diagram showing distinct and shared oscillations for each period. (**G, H**) Heatmap (**G**) and quantification (**H**) of decomposition of raw temporal transcriptome into oscillations cycling at ~16-hour, ~12-hour or ~24-hour periods. (**I**) Representative temporal expression of selective genes and eigenvalue/pencil decomposition uncovering all superimposed oscillations for each gene. (**J**) GO analysis of all genes in **G**.

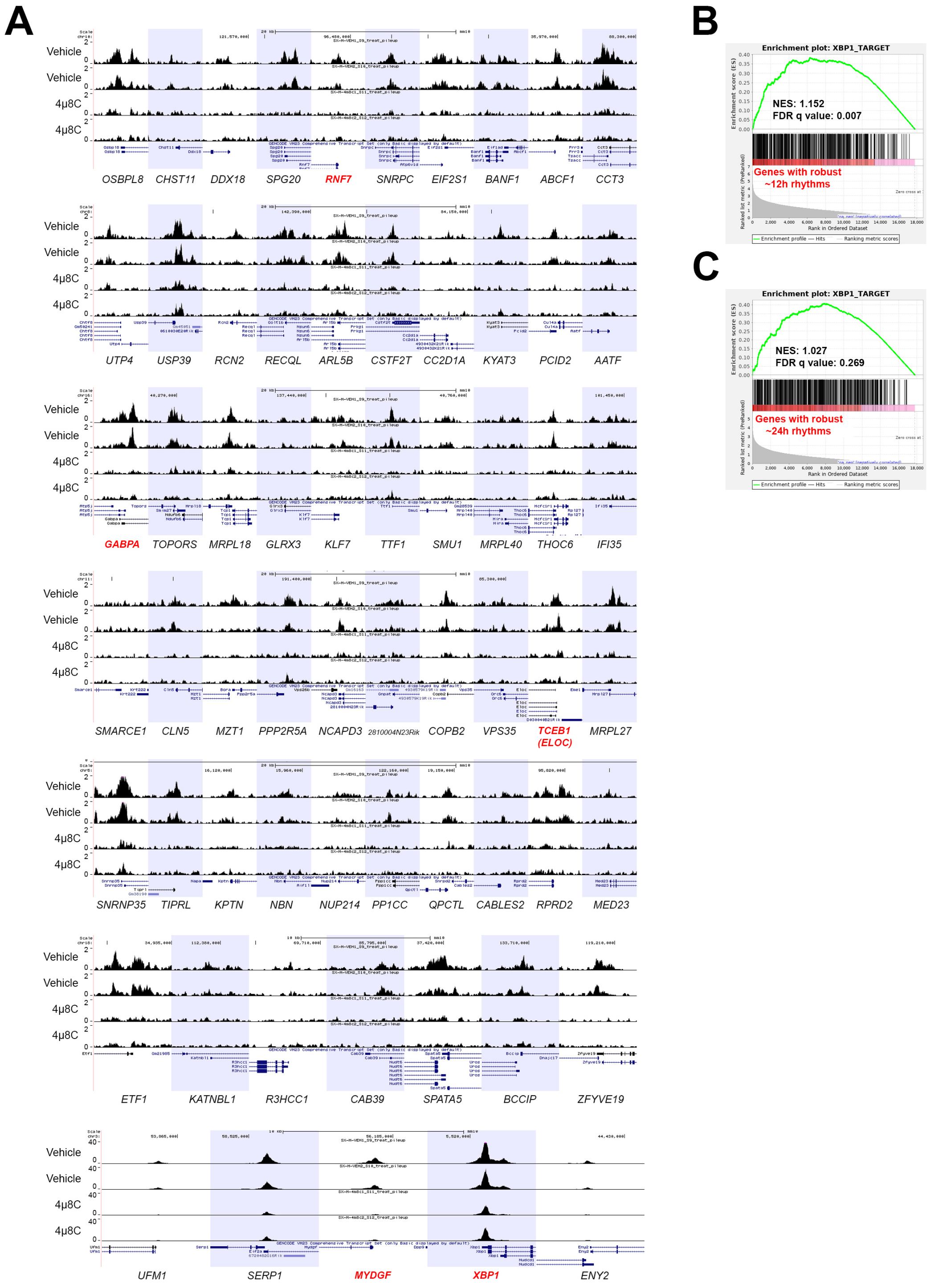

Fig. S8. XBP1 chromatin binding landscape of 12-hour genes in mouse Th2 cells. (**A**) UCSC genome browser view of XBP1 chromatin recruitment to the promoters of a selected set of ~12-hour genes in murine Th2 cells. (**B, C**) GSEA showing enrichment score of top 500 XBP1 target genes in Th2 cells (ranked by fold reduction of target gene expression with XBP1 deletion) in robust ~12-hour (**B**) or ~24-hour (**C**) genes identified in humans.

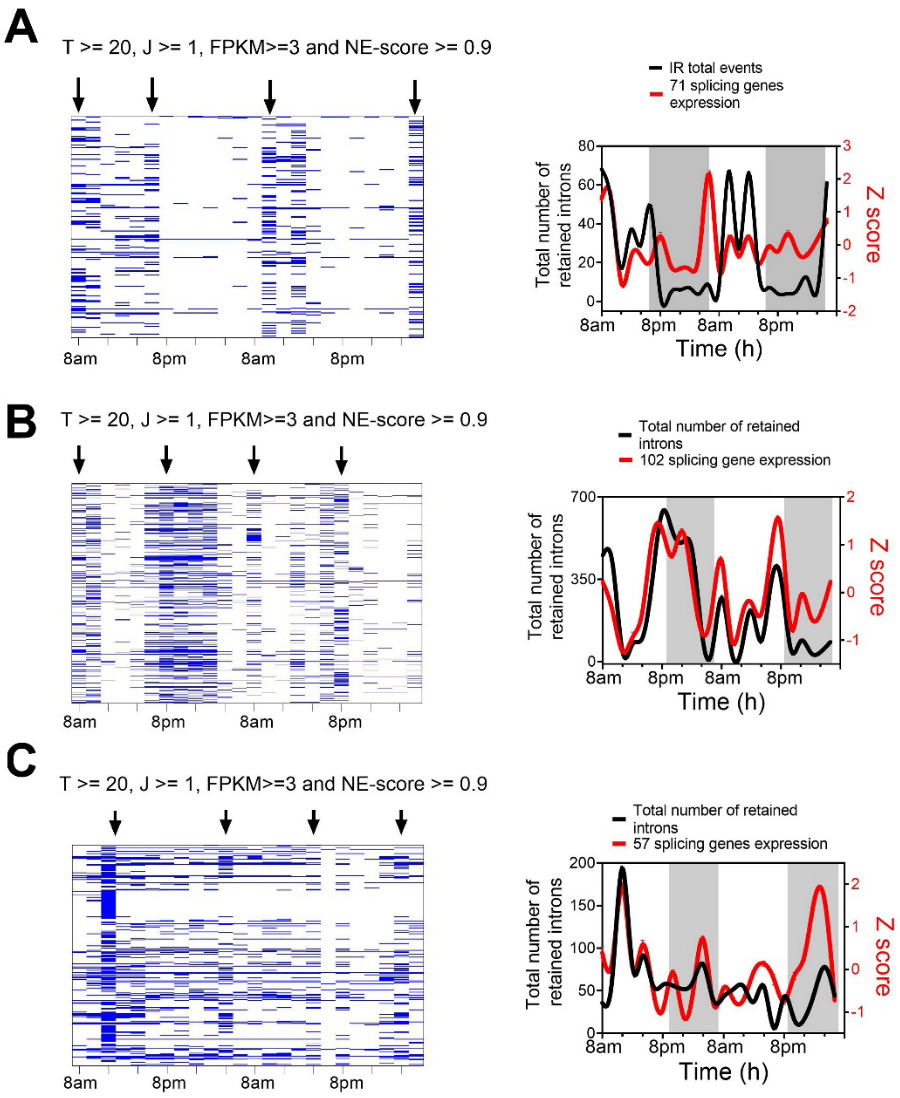

D

	Intron retention events rhythms	
	Period	p value
First	10h	0.2041
Second	12h	0.0439
Third	12h	0.0056
Meta	10-12h	2.990E-03

Fig. S9. ~12-hour rhythms are synchronized to global intron retention rhythms. Criterions for IR are set as T>=20, J>=1, FPKM>=3 and NE score>=0.9. (**A-C**) Heatmap (left) and quantification (right) of temporal IR events, superimposed with the Z score normalized temporal expression of ~12-hour splicing gene expression in the first (**A**), second (**B**) and third (**C**) participants. IR events are detected with the more stringent setting of the iRead algorithm as described in the methods section. (**D**) Statistics for IR ~12-hour rhythms detection by RAIN.

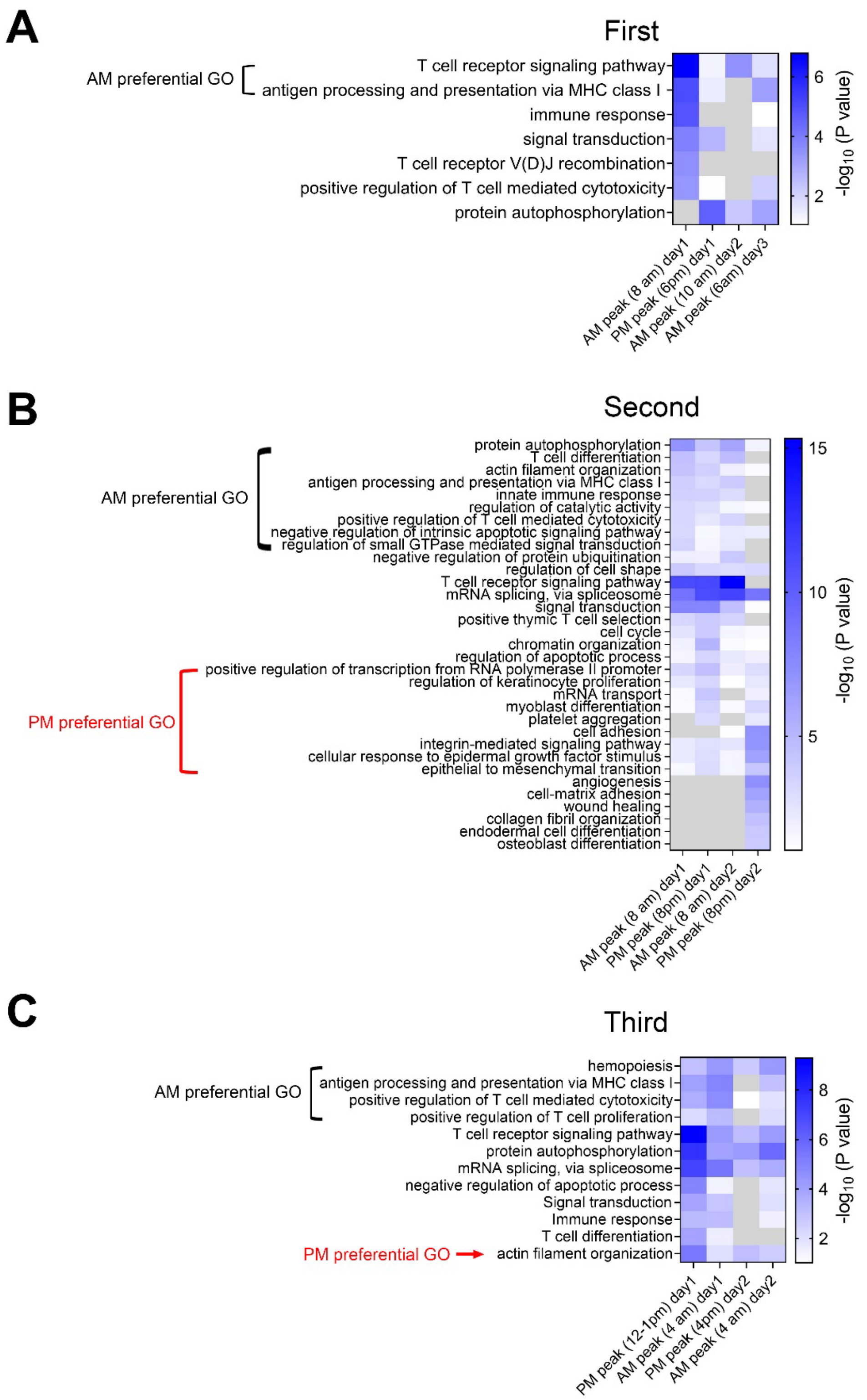

Fig. S10. Genes with intron retentions are enriched in immune genes. Intron retention events are selected with default settings T>=20, J>=1, FPKM>=2 and NE score>=0.9. GO analysis of IR genes at different peaks in the first (**A**), second (**B**) and third (**C**) participant.

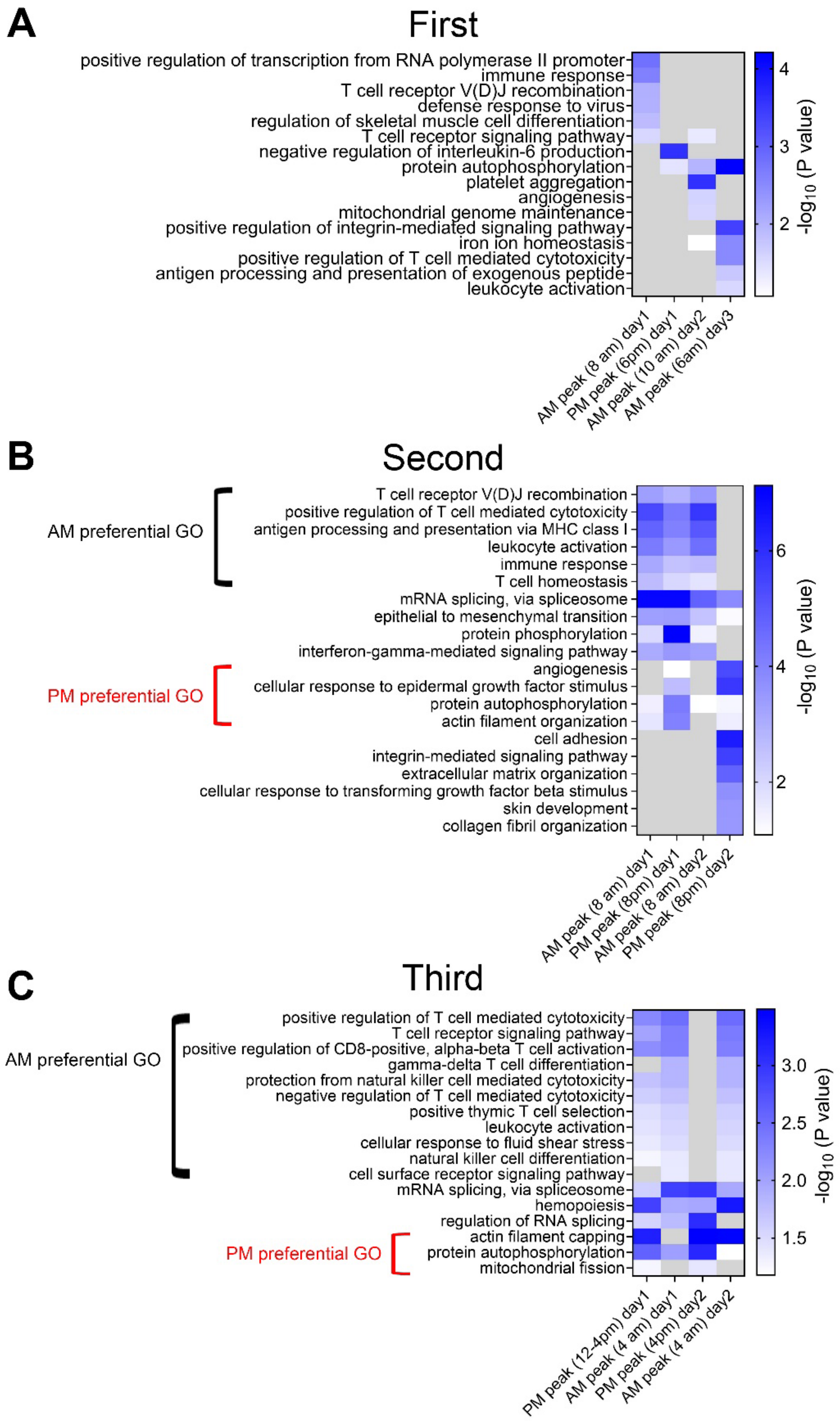

Fig. S11. Genes with intron retentions (more stringent criterion) are enriched in immune genes. Intron retention events are selected with default settings T>=20, J>=1, FPKM>=3 and NE score>=0.9. GO analysis of IR genes at different peaks in the first (**A**), second (**B**) and third (**C**) participant.

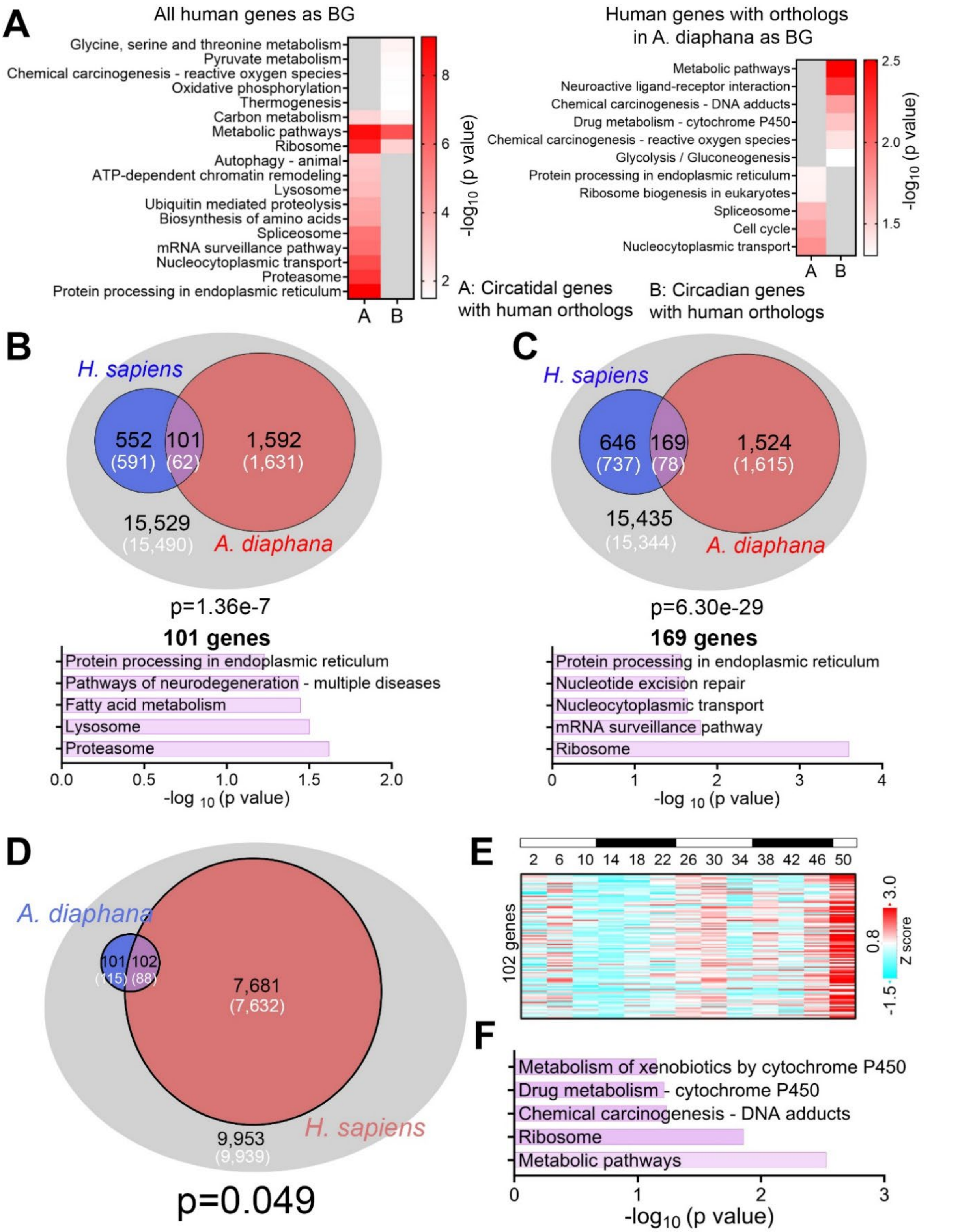

Fig. S12. Evolutionary conservation of ~12-hour, but not ~24-hour gene programs between A. diaphana and human. (**A**) GO analysis of all circatidal genes in A. diaphana that have human orthologs using all human genes (left) or only those with A. diaphana orthologs (right) as

background. (**B, C**) Venn diagram comparing distinct and shared ~12-hour genes in human (653 12-hour genes with meta adj-P<0.05 in **B** and 851 ~12-hour genes reported in Fig. S4B in **C** and *A. diaphana* (reported in [49]). Only genes that are expressed in human white blood cells (denoted by the grey circle) are included in the analysis. Both observed and predicted number of genes (under the null hypothesis that ~12-hour genes are not evolutionarily conserved and thus independently detected in these two species) are further shown. P values are calculated by the Chi-square test. GO analysis showing enriched KEGG terms for the 101 and 169 common genes, respectively. (**D**) Venn diagram comparing distinct and shared ~24-hour genes in human (meta adj-P<0.1) and *A. diaphana* (reported in [49]). Only genes that are expressed in human white blood cells (denoted by the grey circle) are included in the analysis. Both observed and predicted number of genes (under the null hypothesis that ~24-hour genes are not evolutionarily conserved and thus independently detected in these two species) are further shown. P value of 0.049 is calculated by Chi-square test. (**E, F**) Heatmap of temporal expression (Z score normalized) of 102 ~24-hour genes in *A. diaphana* (**E**) and GO analysis of the top enriched pathways (**F**).

Table S1. RPKM quantification of temporal expression for the human participants

Table S2. RAIN results for ~12-hour genes and meta-analysis of ~24-hour genes.

Table S3. Eigenvalue/pencil results
